# Predicting Drug-Disease Associations via Multi-Task Learning Based on Collective Matrix Factorization

**DOI:** 10.3389/fbioe.2020.00218

**Published:** 2020-04-09

**Authors:** Feng Huang, Yang Qiu, Qiaojun Li, Shichao Liu, Fuchuan Ni

**Affiliations:** ^1^College of Informatics, Huazhong Agricultural University, Wuhan, China; ^2^School of Electronic and Information Engineering, Henan Polytechnic Institute, Henan Nanyang, China; ^3^Hubei Engineering Technology Research Center of Agricultural Big Data, Wuhan, China

**Keywords:** drug-disease association, predicting association type, similarity, collective matrix factorization, multi-task learning

## Abstract

Identifying drug-disease associations is integral to drug development. Computationally prioritizing candidate drug-disease associations has attracted growing attention due to its contribution to reducing the cost of laboratory screening. Drug-disease associations involve different association types, such as drug indications and drug side effects. However, the existing models for predicting drug-disease associations merely concentrate on independent tasks: recommending novel indications to benefit drug repositioning, predicting potential side effects to prevent drug-induced risk, or only determining the existence of drug-disease association. They ignore crucial prior knowledge of the correlations between different association types. Since the Comparative Toxicogenomics Database (CTD) annotates the drug-disease associations as therapeutic or marker/mechanism, we consider predicting the two types of association. To this end, we propose a collective matrix factorization-based multi-task learning method (CMFMTL) in this paper. CMFMTL handles the problem as multi-task learning where each task is to predict one type of association, and two tasks complement and improve each other by capturing the relatedness between them. First, drug-disease associations are represented as a bipartite network with two types of links representing therapeutic effects and non-therapeutic effects. Then, CMFMTL, respectively, approximates the association matrix regarding each link type by matrix tri-factorization, and shares the low-dimensional latent representations for drugs and diseases in the two related tasks for the goal of collective learning. Finally, CMFMTL puts the two tasks into a unified framework and an efficient algorithm is developed to solve our proposed optimization problem. In the computational experiments, CMFMTL outperforms several state-of-the-art methods both in the two tasks. Moreover, case studies show that CMFMTL helps to find out novel drug-disease associations that are not included in CTD, and simultaneously predicts their association types.

## Introduction

Drugs are chemicals used to treat, cure, prevent, or diagnose diseases. The development of a new drug has three steps: discovery stage, preclinical stage, and clinical stage (Wilson, 2006), which takes about 15 years (Dimasi, 2001) and costs about 1,000 million U.S. dollars (Adams and Brantner, 2006). Such a costly and time-consuming process remains at huge risk. After marketing, approved drugs will be surveilled to reassess their safety and some side effects may be reported (Liang et al., 2019). If adverse drug reactions cause serious consequences then the drugs are taken off the shelves and approval is withdrawn, bringing enormous economic loss to pharmaceutical companies. Therefore, identifying drug-disease associations is of significant importance. On one hand, finding novel indications for drugs can be helpful for more effective drug development. On the other hand, screening potential side effects for drugs can reduce the risk of medicine use. But traditional wet-lab experiments are expensive and laborious. In light of these challenges, computational methods which associate drugs with diseases have attracted growing attention from the biomedical community.

Recently, a large number of computational methods have been proposed for the drug-disease association prediction. Gottlieb et al. (2011) constructed a drug-disease association classifier based on the integration of drug molecular structures, drug molecular activities, and disease semantic information. Pauwels et al. (2011) put chemical structures of drugs in four machine-learning models to train classifiers. Huang et al. (2013) used the random walk to infer the unobserved links in a heterogeneous network merging drugs, genomic information, and disease phenotypes. Cheng et al. (2013) adopted a resource allocation-based approach to infer unobserved side effects for existing drugs. Oh et al. (2014) extracted features representing drug-disease associations by using similarity-based features and module-distance-based features, and then, respectively, adopted decision tree, multi-layer perception, and random forest to build prediction models. Wang et al. (2014) designed a computational framework based on a three-layer heterogeneous network model (TL-HGBI). Zhang et al. proposed the multi-label learning method (Zhang et al., 2015), and the linear neighborhood similarity-based method (Zhang et al., 2016a, 2017c) for side effect prediction. Moghadam et al. (2016) adopted the kernel fusion technique to combine different drug features and disease features, and then built SVM models. Liang et al. (2017) proposed a Laplacian regularized sparse subspace learning method (LRSSL) which integrated drug chemical structures, drug target domains, and target ontology. Zhang et al. (2016b) defined this task as the recommender problem, and introduced restricted Boltzmann machine and collaborative filtering to predict unobserved side effects. Luo et al. (2018) designed a drug repositioning recommendation system (DRRS) and used a matrix completion algorithm to fill out the unknown entries in drug-disease associations. Zhang et al. (2017b) presented a novel bipartite network-based method, which only used known drug-disease associations to predict unobserved associations. Zhang et al. (2018c) proposed a similarly constrained matrix factorization method, which utilized known drug-disease associations, drug features, and disease semantic information. Xuan et al. (2019) proposed a computational drug repositioning method through the integration of multiple drug similarity and disease similarity.

The existing models for predicting drug-disease associations only focus on indication prediction or side effect prediction, but ignore the relatedness of the two tasks, which is vital for knowledge of drug-disease associations. Despite the fact that some studies (Yang and Agarwal, 2011; Wang et al., 2013) considered drug side effects as auxiliary information for indication prediction, they failed to comprehensively make use of prior knowledge. According to the Comparative Toxicogenomics Database (CTD) (Davis et al., 2013, 2017) some drugs have therapeutic effects on diseases, e.g., sorafenib is usually used to treat leukemia (Auclair et al., 2007). Some drugs play a role in the etiology of diseases which can be regarded as side effects, biomarkers or other effects, e.g., increased sediment in the brain of amyloid beta-protein may correlate with Alzheimer's disease (Yamada et al., 2008), continued exposure to nicotine may cause lung cancer, and over-dose ingestion of caffeine may lead to a headache. Almost all drugs exert only one type of effect on a certain disease. In general, if a drug can be used to treat a disease then one can know that the drug is much less likely to exert other effects on the disease. Hence, predicting two types of drug-disease associations requires multi-task learning with two closely related tasks, where each task is meant to predict one type of association. It is a natural foresight that addressing the two tasks in one uniform framework can make them complement each other. To this end, we devise a model for capturing the relatedness between the tasks and retaining the individuality of each of them.

In this paper, we propose a collective matrix factorization-based multi-task learning method (abbreviated as “CMFMTL”) to predict two types of drug-disease associations. From the CTD database, we collect drug-disease associations annotated as therapeutic or marker/mechanism (non-therapeutic), and then construct a drug-disease network with two types of links representing therapeutic effects and non-therapeutic effects. CMFMTL, respectively, approximates the association matrix regarding each link type by matrix tri-factorization, and shares the low-dimensional latent representations for drugs and diseases in the two related tasks for the goal of collective learning. We also develop an efficient algorithm to solve our proposed model. In the computational experiments, CMFMTL outperforms several state-of-the-art methods in both tasks. Moreover, case studies show that CMFMTL helps to find out novel drug-disease associations that are not included in CTD, and simultaneously predicts their association types.

## Materials and Methods

### Dataset

The Comparative Toxicogenomics Database (CTD) (Davis et al., 2013, 2017) is a publicly available database that intends to advance understanding about how environmental exposures affect human health. Zhang et al. (2018c) downloaded the chemical-disease associations from the CTD. Then they mapped the chemicals into the DrugBank (Knox et al., 2011; Law et al., 2014; Wishart et al., 2018) database, a comprehensive knowledge base for drugs, to obtain approved drugs and some biological features for drugs, such as chemical substructures, targets, enzymes, pathways, and drug-drug interactions. The diseases were matched into the Medical Subject Headings (MeSH), a vocabulary thesaurus for biomedicine controlled by the National Library of Medicine, to collect the MeSH descriptors of diseases for the use of calculating disease semantic similarity. We use this dataset as our benchmark dataset to evaluate the performance of models.

As we described above, chemical-disease associations in CTD are annotated as therapeutic or marker/mechanism. Therapeutic associations mean that chemicals play a therapeutic role in diseases, while marker/mechanism associations mean that chemicals correlate with diseases. In this study, we can easily label these associations as therapeutic associations or non-therapeutic (marker/mechanism) associations. Extremely few associations are simultaneously annotated as two association types. Without loss of statistical properties of the data, we only label the extreme cases as therapeutic associations. Finally, the benchmark dataset contains 18,416 drug-disease associations involving 269 drugs and 598 diseases. Among these associations, 6,244 associations are therapeutic associations and 12,172 associations are non-therapeutic associations.

### Similarities for Drugs and Diseases

Let $R=\left\{r_{1},r_{2},\ldots ,r_{m}\right\}$ denote the set of drugs and $D=\left\{d_{1},d_{2},\ldots ,d_{n}\right\}$ represent the set of diseases. In this section, we introduce the drug-drug similarity and the disease-disease semantic similarity.

### Drug-Drug Similarity

A feature of a drug is a collection of entities or attributes related to the drug. Thus, we can use the Tanimoto score (Tanimoto, 1958) [also known as Jaccard index (Jaccard, 1908) for measurement of similarity between two sets] to calculate the drug-drug similarity. Let Γ_*i*_ and Γ_*j*_ denote features of two drugs, the Jaccard index is described as:

(1) $$\begin{array}{r} S_{Jaccard}\left(\Gamma_{i},\Gamma_{j}\right)=\frac{\left|\Gamma_{i}\cap \Gamma_{j}\right|}{\left|\Gamma_{i}\cup \Gamma_{j}\right|}=\frac{\left|\Gamma_{i}\cap \Gamma_{j}\right|}{\left|\Gamma_{i}|+|\Gamma_{j}|-|\Gamma_{i}\cap \Gamma_{j}\right|} \end{array}$$

where |·| is the number of elements in the set.

Let $\Gamma ={⋃}_{i}^{m}\Gamma_{i}$ represent the union set of features of *m* drugs and |Γ| = *c*, and then the drug feature can be encoded as a *c*-dimensionality binary vector, e.g., the drug *r*_*i*_ is encoded as $x_{i}\in {\left\{0,1\right\}}^{c}$ where the *i*th element is set to 1 if the *i*th descriptor in Γ belongs to the set Γ_*i*_; otherwise, it is set to zero. Obviously, the Equation (1) can be rewritten as:

(2) $$\begin{array}{r} S_{drug}\left(r_{i},r_{j}\right)=\frac{\langle x_{i},x_{j}\rangle }{\langle x_{i},e\rangle +\langle x_{j},e\rangle -\langle x_{i},x_{j}\rangle } \end{array}$$

where 〈·, ·〉 is the inner product of two vectors and *e* is a vector with all elements equal to 1.

### Disease-Disease Similarity

As described in Wang et al. (2010) Gong et al. (2019), Zhang et al. (2019b), the hierarchical MeSH descriptors of diseases can be compiled as Directed Acyclic Graphs (DAGs), where vertexes represent the diseases and edges represent the relationships between different diseases. For a disease *d*, the DAG is denoted as *DAG*_*d*_ = (*V*_*d*_, *E*_*d*_), where *V*_*d*_ is the set of all ancestors of *d* (including itself) and *E*_*d*_ is the set of links from ancestor disease to their children. The semantic contribution of disease *t* ∈ *V*_*d*_ to disease *d* is defined as:

(3) $$\begin{array}{r} SC_{d}\left(t\right)=\{\begin{array}{c} \;1\;if\;t=d\\ max\left\{\;\Delta \times SC_{d}\left(t^{\prime }\right)|t^{\prime }\in C\left(t\right)\right\}\;if\;t\neq d \end{array} \end{array}$$

where *C*(*t*) is the set of children nodes of *t*, is the semantic contribution factor. Then the semantic value of disease *d* is calculated by:

(4) $$\begin{array}{r} SV_{d}=\sum t\in V_{d}SC_{d}\left(t\right) \end{array}$$

Finally, the semantic similarity between two diseases *d*_*i*_ and *d*_*j*_ is calculated by:

(5) $$\begin{array}{r} S_{disease}\left(d_{i},d_{j}\right)=\frac{\sum_{t\in V_{d_{i}}\cap V_{d_{j}}}\left(SC_{d_{i}}\left(t\right)+SC_{d_{j}}\left(t\right)\right)}{SV_{d_{i}}+SV_{d_{j}}} \end{array}$$

### Collective Matrix Factorization-Based Multi-Task Learning Method

#### Multi-Task Learning

Multi-task learning is an inductive transfer learning approach that captures the connections amongst multiple related learning tasks as an inductive bias by a specific shared mechanism (Ando and Zhang, 2005), and exploits the task relatedness as prior knowledge to improve generalization capabilities (Caruana, 1997). During the learning process of multi-task learning, these related tasks are learned in parallel and complement each other, which is saying that what is learned for each task can help other tasks be learned better. In this work, we formulate predicting drug-disease therapeutic associations and non-therapeutic associations as two related tasks and put them in a multi-task setting for better predictive performance.

#### Overview

**Figure 1:**
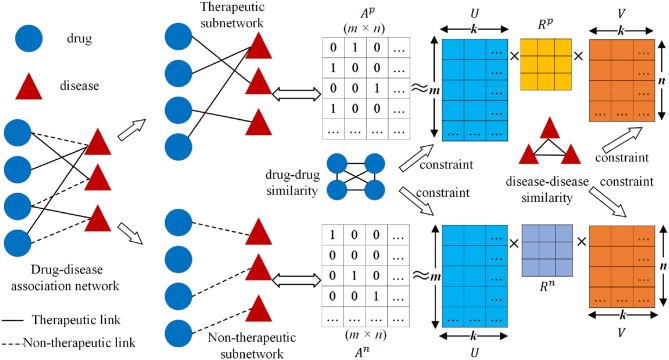
Workflow of collective matrix factorization-based multi-task learning method (CMFMTL): *A*^*p*^ is the corresponding binary matrix for the therapeutic subnetwork; *A*^*n*^ is the corresponding binary matrix for non-therapeutic subnetwork; *U* ∈ ℝ^*m*×*k*^ and *V* ∈ ℝ^*n*×*k*^ are, respectively, the low-dimensional representations for drugs and diseases; *R*^*p*^ and *R*^*n*^ are coefficient matrices.

The workflow of the collective matrix factorization-based multi-task learning method (CMFMTL) is demonstrated in Figure 1. The CMFMTL involves several critical steps to construct a prediction model for predicting two types of drug-disease associations. First, a drug-disease association network is constructed based on known associations and their types: therapeutic and non-therapeutic. Second, the drug-disease association network is divided into two subnetworks: one subnetwork involves links representing therapeutic associations and the other contains links representing non-therapeutic associations. Third, two binary matrices regarding the two subnetworks are simultaneously factorized into the product of three low-dimensional matrices which are served as latent components for drugs and diseases, and coefficient matrices measuring the level of interaction between latent components. The latent representations of drugs and diseases are shared in the two factorization tasks for capturing the relatedness of these two tasks, and the different coefficient matrices maintain the specificity of two tasks. Finally, the graph Laplacian regularizations (Cai et al., 2011) based on the biological features of drugs and diseases are introduced to further enhance interpretability and generalization.

#### Objective Function of CMFMTL

Given a set of drugs $R=\left\{r_{1},r_{2},\ldots ,r_{m}\right\}$ and a set of diseases $D=\left\{d_{1},d_{2},\ldots ,d_{n}\right\}$, we can construct a relation network G, which uses R and D as two disjoint sets of nodes. There are two types of links between nodes in R and nodes in D. The link between drug *r*_*i*_ and disease *d*_*j*_ is labeled as therapeutic link if the drug *r*_*i*_ has a therapeutic effect on the disease *d*_*j*_; the edge is labeled as a non-therapeutic link if the drug *r*_*i*_ has a non-therapeutic effect on the disease *d*_*j*_. Then the drug-disease association network G can be divided into a therapeutic subnetwork $G_{p}$ and a non-therapeutic subnetwork $G_{n}$. *A*^*p*^ ∈ {0, 1}^*m*×*n*^ is the corresponding binary matrix for $G_{p}$, where $A_{ij}^{p}=1$ if the drug *r*_*i*_ has a therapeutic link to the disease *d*_*j*_, otherwise $A_{ij}^{p}=0$. Similarly, *A*^*n*^ ∈ {0, 1}^*m*×*n*^ is the corresponding binary matrix for $G_{n}$, where $A_{ij}^{n}=1$ if the drug *r*_*i*_ has a non-therapeutic link to the disease *d*_*j*_, otherwise $A_{ij}^{n}=0$. We employ the matrix tri-factorization technique to model *A*^*p*^ and *A*^*n*^, respectively, and map the drugs (diseases) into common latent representations shared in two tasks. Specifically, we approximate the association matrices *A*^*p*^ and *A*^*n*^ by minimizing the reconstruction errors:

(6) $$\begin{array}{r} \underset{U,V,R^{P},R^{n}}{min}\frac{1}{2}\left(||A^{p}-UR^{p}V^{T}|{|}_{F}^{2}+||A^{n}-UR^{n}V^{T}|{|}_{F}^{2}\right) \end{array}$$

where $||\cdot |{|}_{F}^{2}$ is the Frobenius norm; *U* ∈ ℝ^*m*×*k*^ and *V* ∈ ℝ^*n*×*k*^ are the low-dimensional representations for drugs and diseases, respectively; *R*^*p*^ and *R*^*n*^ are coefficient matrices which model how the latent representations interact in the respective association type; *k* < min(*m, n*) is the dimensionality of the low-dimensional space.

Since Equation (6) maps drugs and diseases into a low-dimensional space, a natural idea occurs that the low-dimensional representations should preserve the underlying interconnection information of drugs and diseases. Studies on manifold learning (Belkin et al., 2006; Ma and Fu, 2012; Zhang et al., 2018a), spectral graph theory (Chung, 1997; Rana et al., 2015) and their applications (Zhang et al., 2016a, 2017a,b,c, 2018b; Ruan et al., 2017) have shown that the learning performance can be signally enhanced, if the local topological invariant properties are preserved. Cai et al. (2011) proposed Laplacian regularizations to achieve this goal. Here, we introduce the regularization terms based on biological features about drugs and diseases to incorporate similarity information in our model. We denote the drug-drug similarity matrix as *W*^*r*^ ∈ ℝ^*m*×*m*^ where the (*i, j*)th entry $w_{ij}^{r}=S_{drug}\left(r_{i},r_{j}\right)$ and the disease-disease semantic similarity as *W*^*d*^ ∈ ℝ^*n*×*n*^ where the (*i, j*)th entry $w_{ij}^{d}=S_{disease}\left(d_{i},d_{j}\right)$, which are previously calculated in Equations (2) and (5). Then, the graph Laplacian matrices are constructed as *L*^*U*^ = *D*^*r*^ − *W*^*r*^ and *L*^*V*^ = *D*^*d*^ − *W*^*d*^, where *D*^*r*^ and *D*^*d*^ are, respectively, diagonal matrices whose diagonal elements are corresponding row sums of *W*^*r*^ and *W*^*d*^. The graph Laplacian regularizations are formulated as:

(7) $$\begin{array}{r} R_{1}=tr\left(U^{T}L^{U}U\right)=\frac{1}{2}\overset{m}{\underset{i,j=1}{\sum }}||U\left(i,:\right)-U\left(j,:\right)|{|}_{2}^{2}w_{ij}^{r}\\ R_{2}=tr\left(V^{T}L^{V}V\right)=\frac{1}{2}\overset{n}{\underset{i,j=1}{\sum }}||V\left(i,:\right)-V\left(j,:\right)|{|}_{2}^{2}w_{ij}^{d} \end{array}$$

where *tr*(·) denotes the trace of a square matrix; *U*(*i*, :) and *V*(*i*, :) are the *i*th row vector of *U* and *V*, respectively; more details for the second equality can be referred to in Cai et al. (2011). Obviously, minimizing $R_{1}$ (or $R_{2}$) will lead to a result that the drug *r*_*i*_ (the disease *d*_*i*_) is closer to the drug *r*_*j*_ (the disease *d*_*j*_) in the low-dimensional space if the similarity between them $w_{ij}^{r}$ ($w_{ij}^{d}$) is higher. Additionally, we introduce the *L*_2_ regularizations to reinforce the smoothness of *U*, *V*, *R*^*p*^, and *R*^*n*^. Therefore, we obtain the optimization objective of the CMFMTL by combining the *L*_2_ regularizations, Equations (6) and (7):

(8) $$\begin{array}{r} \underset{U,V,R^{P},R^{n}}{min}\frac{1}{2}\left(||A^{p}-UR^{p}V^{T}|{|}_{F}^{2}+||A^{n}-UR^{n}V^{T}|{|}_{F}^{2}\right)\\ +\frac{\alpha }{2}tr\left(U^{T}L^{U}U\right)+\frac{\beta }{2}tr\left(V^{T}L^{V}V\right)\\ +\frac{\lambda }{2}\left(||U|{|}_{F}^{2}+||V|{|}_{F}^{2}+||R^{p}|{|}_{F}^{2}+||R^{n}|{|}_{F}^{2}\right) \end{array}$$

where α, β and λ are the regularization parameters.

### Optimization

To efficiently solve problem (8), we equivalently convert it into an equation constrained optimization problem:

(9) $$\begin{array}{r} \underset{U,V,R^{P},R^{n}}{min}\frac{1}{2}\left(||A^{p}-UR^{p}V^{T}|{|}_{F}^{2}+||A^{n}-UR^{n}V^{T}|{|}_{F}^{2}\right)\\ +\frac{\alpha }{2}tr\left(W^{T}L^{U}W\right)+\frac{\beta }{2}tr\left(J^{T}L^{V}J\right)\\ +\frac{\lambda }{2}\left(||U|{|}_{F}^{2}+||V|{|}_{F}^{2}+||R^{p}|{|}_{F}^{2}+||R^{n}|{|}_{F}^{2}\right)\\ \;s.t.\;J=V,\;W=U \end{array}$$

Then, the augmented Lagrangian function L of Equation (9) is introduced as follows:

(10) $$\begin{array}{l} ℒ=\frac{1}{2}\left({\left\|A^{p}-UR^{p}V^{T}\right\|}_{F}^{2}+{\left\|A^{n}-UR^{n}V^{T}\right\|}_{F}^{2}\right)\\ \;+\frac{\alpha }{2}tr\left(W^{T}L^{U}W\right)+\frac{\beta }{2}tr\left(J^{T}L^{V}J\right)+\frac{\lambda }{2}({\left\|U\right\|}_{F}^{2}+{\left\|V\right\|}_{F}^{2}\\ \;+{\left\|R^{p}\right\|}_{F}^{2}+{\left\|R^{n}\right\|}_{F}^{2})+tr\left(Z^{T}\left(W-U\right)\right)\\ \;+\frac{\rho_{1}}{2}{\left\|W-U\right\|}_{F}^{2}+tr\left(Y^{T}\left(J-V\right)\right)+\frac{\rho_{2}}{2}{\left\|J-V\right\|}_{F}^{2} \end{array}$$

where *J* and *W* are the auxiliary variables; ρ_1_ > 0, ρ_2_ > 0 are called as the penalty parameters; *Z* and *Y* are the Lagrange multipliers. We resort to the alternating direction method of multipliers (ADMM) framework (Boyd et al., 2011) to devise an alternately updating rule for optimizing Equation (10).

Next, differentiating L with respect to *J*, *W*, *U*, and *V*, respectively, and setting the partial derivatives to zero, we have the following updating rule:

(11) $$\begin{array}{l} J={\left(\beta L^{V}+\rho_{2}I\right)}^{-1}\left(\rho_{2}V-Y\right)\\ W={\left(\alpha L^{U}+\rho_{1}I\right)}^{-1}\left(\rho_{1}U-Z\right)\\ U=\left(A^{p}VR^{p}{}^{T}+A^{n}VR^{n}{}^{T}+Z+\rho_{1}W\right)(R^{p}V^{T}VR^{p}{}^{T}\\ \;{+\;R^{n}V^{T}VR^{n}{}^{T}+\lambda I+\rho_{1}I)}^{-1}\\ V=\left(A^{p}{}^{T}UR^{p}+A^{n}{}^{T}UR^{n}+Y+\rho_{2}J\right)({\left(UR^{p}\right)}^{T}UR^{p}\\ \;{+{\left(UR^{n}\right)}^{T}UR^{n}+\lambda I+\rho_{2}I)}^{-1} \end{array}$$

where *I* represents the identity matrix with an adaptive dimensionality in different equations. When fixing other variables, the objective function for *R*^*p*^ is simplified as:

(12) $$\begin{array}{r} \underset{R^{P}}{min}\frac{1}{2}||A^{p}-UR^{p}V^{T}|{|}_{F}^{2}+\frac{\lambda }{2}||R^{p}|{|}_{F}^{2} \end{array}$$

Equation (12) can be efficaciously solved by the algorithm proposed in Yu et al. (2014) which leverages the conjugate gradient method (CG) to improve the efficiency of the solver. Here, we omit the details about the algorithm, and denote the solution for the Equation (12) solved by the algorithm as *CG*(*R*^*p*^). The objective function with regard to *R*^*n*^ shares the same optimization structure with the Equation (12), and thus we denote the solution as *CG*(*R*^*n*^).

Finally, the Lagrange multipliers and the penalty parameter are updated as follows:

(13) $$\begin{array}{rcl} Y & = & Y+\rho_{2}\left(J-V\right)\\ Z & = & Z+\rho_{1}\left(W-U\right)\\ \rho_{1} & = & \mu \rho_{1}\\ \rho_{2} & = & \mu \rho_{2} \end{array}$$

We alternatively update all variables until convergence and the whole process are summarized in Algorithm 1. According to Yu et al. (2014), the main operation in each iteration of the conjugate gradient procedure is a multiplication of three matrices, which can be done in *O*(min(*m, n*)*k*^2^ + *mnk* + *k*^3^) time. We set the maximal iterative number in conjugate gradient procedure as *t*. In each iteration of ADMM, the main operations contain several matrix inverse calculations [in Equation (11)] that cost *O*(*n*^3^ + *m*^3^ + *k*^3^), several matrix multiplications [in Equation (11) and the initialization for conjugate gradient procedure] that cost *O*(*n*^2^*k* + *m*^2^*k* + *mk*^2^ + *nk*^2^ + *k*^3^ + *mnk*) and the conjugate gradient procedure that cost *O*((min(*m, n*)*k*^2^ + *mnk* + *k*^3^)*t*).

**Algorithm 1:** The updated process of CMFMTL.

**Input:** known drug-disease therapeutic association matrix, *A*^*p*^ ∈ {0, 1}^*m*×*n*^; known drug-disease non-therapeutic association matrix, *A*^*n*^ ∈ {0, 1}^*m*×*n*^; drug similarity matrix, *w*^*r*^ ∈ ℝ^*m*×*m*^; disease similarity matrix, *w*^*d*^ ∈ ℝ^*n*×*n*^; dimensionality of the embedded space, *k* < min(*m, n*); regularization parameters, α > 0, β > 0 and λ> 0 **Output:** the prediction matrices ^*A*^p^*^, ^*A*^n^*^ **Initialize** *V* ∈ ℝ^*n*×*k*^ and *U* ∈ ℝ^*m*×*k*^ in the interval [0, 1] randomly; *Y* = 0 and *Z* = 0; ρ_1_ = ρ_2_ = 1 **Repeat** **Update** *R*^*p*^ and *R*^*n*^ using *R*^*p*^ = *CG*(*R*^*p*^), *R*^*n*^ = *CG*(*R*^*n*^) **Update** *J*, *W*, *U* and *V* via the equation (11) **Update** *Y*, *Z*, ρ_1_ and ρ_2_ via the equation (13) **End until convergence** **Output** ^*A*^p^*^, ^*A*^n^*^ using ^*A*^p^*^ = *UR*^*p*^*V*^*T*^,^*A*^n^*^ = *UR*^*n*^*V*^*T*^

## Results and Discussion

### Evaluation Metrics

In our experiment, 5-fold cross validation (5-CV) experiments are conducted to systematically evaluate prediction models. Considering assessing models in two tasks, where predicting drug-disease therapeutic associations is called task 1 and the other is called task 2, we respectively split known therapeutic associations and non-therapeutic associations into five equal-sized parts at random. In each task, one of the five subsets is considered as the testing set in turn, and the remaining four subsets are combined as the training set. The metrices can be calculated in each fold, and the average of five evaluations is adopted.

Several evaluation metrics, such as sensitivity (SE, also known as recall), specificity (SP), accuracy (ACC), precision (PRE), and F-measure (F), are calculated. Since they depend on a threshold to classify predictions as positive or negative, we adopt the threshold which produces the max F-measure. Moreover, the area under the receiver-operating characteristic curve (AUC) and the area under the precision-recall curve (AUPR) are adopted as the primary metrics.

### Parameter Setting

**Figure 2:**
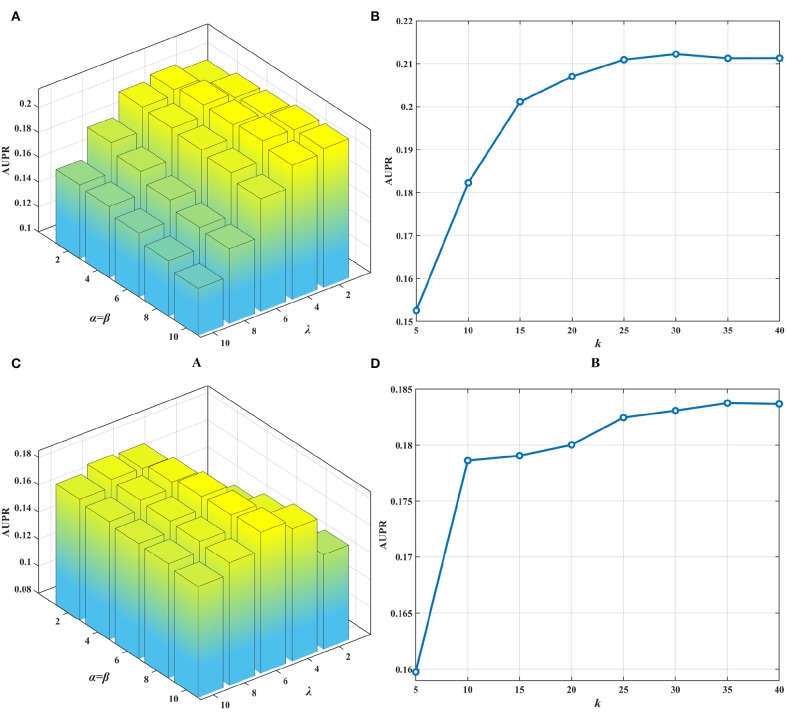
Influence of parameters on the performance of CMFMTL involving two tasks: **(A)** shows the influence of α, β, λ on the AUPR score in task 1. **(B)** indicates the effect of *k* on the AUPR score in task 1. **(C)** illustrates the impact of α, β, λ on the AUPR score in task 2. **(D)** demonstrates the perturbation of *k* on the AUPR score in task 2.

The collective matrix factorization-based multi-task learning method (CMFMTL) has four key parameters: the dimensionality of the common latent space *k*, and the regularization coefficients α, β, and λ. These parameters may have great impact on the performances of the CMFMTL, so analysis of parameters is necessary. For simplicity, we set α = β, λ ∈ {2, 4, 6, 8, 10} and *k* ∈ {5, 10, 15, 20, 25, 30, 35, 40}. Note that we have several kinds of drug features as mentioned in section Dataset. We use drug substructures to calculate drug-drug similarity for better performance. For the calculation of disease-disease similarity, we set semantic contribution factor = 0.5 (Zhang et al., 2019b). For the growth factor μ of the penalty parameters ρ_1_ and ρ_2_ in Equation (13), we set μ = 1.1. By grid-search, we obtain the best results with an AUPR of 0.2122 in task 1 when α = β = 8, λ = 4 and *k* = 30; and with an AUPR of 0.1838 in task 2 when α = β = 10, λ = 6 and *k* = 35. Figures 2A,C show the influence of regularization coefficients on the performance of the CMFMTL in task 1 and task 2, respectively. Figures 2B,D correspond to the impact of dimensionality in the two tasks. From some observations, the *L*_2_ regularization coefficient λ may control the trade-off between the two tasks, e.g., greater λ produces better performance in task 2 than task 1. When the dimensionality *k* is too low, models perform poorly. The cause may be that vital data information fails to be fully embedded in the latent representations.

### Comparison With State-of-the-Art Association Prediction Methods

As we discussed above, CMFMTL is a multi-task learning method that simultaneously predicts therapeutic and non-therapeutic associations between drugs and diseases. Existing methods only predict a certain type of drug-disease associations, such as drug indications and side effects. For this reason, we conduct each of several association prediction methods, respectively, on two tasks, and then compare the performance of them with our proposed CMFMTL model.

Here, we consider three state-of-the-art association prediction methods: TL-HGBI, LRSSL, and DRRS, which are the classic or latest works of predicting drug-disease associations. TL-HGBI (Wang et al., 2014) bridged drugs to targets and linked them to diseases to depict a three-layer heterogeneous network. Then, a similarity-based information diffusion method was used to estimate the probabilities of unknown drug-disease associations. LRSSL (Liang et al., 2017) modeled the prediction of drug indications as a joint optimization problem by combining Laplacian regularization with a sparse learning framework, and then an iteratively updating algorithm was implemented to obtain a locally optimal solution. DRRS (Luo et al., 2018) stated drug repositioning as a recommendation problem and utilized a matrix completion algorithm on a block matrix which was concatenated by a drug-disease association matrix, a drug-drug similarity matrix, and a disease-disease similarity matrix. In addition, we use a reduced version of our model (CMFMTL-R) as a baseline method with only one matrix tri-factorization term in Equation (8). CMFMTL-R is a single-task version of CMFMTL, which acquires the result in each task by separately factorizing each corresponding data matrix, e.g., factorizing *A*^*p*^ without decomposing *A*^*n*^ in task 1. We also retain the graph regularizations and *L*_2_ regularizations, and use the same algorithm and parameter setting in CMFMTL-R as in CMFMTL for fair comparison.

All methods are evaluated by 5-CV, and results are shown in Tables 1, 2. Clearly, CMFMTL produces better results than TL-HGBI, LRSSL, and DRRS in the two tasks. It is observed that TL-HGBI and LRSSL perform poorly on our dataset. The most possible reason is that these models are unstable and the performances of them highly rely on their datasets. DRRS is a matrix completion method, which is thought to be able to obtain better results on sparse data. Thereby, DRRS performs better on fewer therapeutic associations than on denser non-therapeutic associations. In contrast, CMFMTL-R performs more steadily in two tasks. Compared with other methods, CMFMTL successfully makes use of all useful association information by collaboratively learning from two tasks. Such advantages make CMFMTL generally outperform other single-task learning methods.

**Table 1:** Performances of Prediction Models in Task 1.

**Methods**	**AUPR**	**AUC**	**SE**	**SP**	**PRE**	**ACC**	**F**
CMFMTL	0.2122	0.8898	0.2888	0.9926	0.2544	0.9866	0.2690
CMFMTL-R	0.1217	0.8543	0.2135	0.9905	0.1644	0.9839	0.1849
TL-HGBI	0.0444	0.7444	0.1265	0.9827	0.0624	0.9753	0.0808
LRSSL	0.0420	0.7341	0.1489	0.9745	0.0490	0.9674	0.0731
DRRS	0.1735	0.8893	0.2756	0.9917	0.2292	0.9856	0.2468

**Table 2:** Performances of Prediction Models in Task 2.

**Methods**	**AUPR**	**AUC**	**SE**	**SP**	**PRE**	**ACC**	**F**
CMFMTL	0.1838	0.8661	0.3091	0.9798	0.2091	0.9686	0.2473
CMFMTL-R	0.1465	0.8449	0.2623	0.9798	0.1812	0.9679	0.2139
TL-HGBI	0.0635	0.7469	0.1839	0.9653	0.0840	0.9523	0.1140
LRSSL	0.0606	0.7393	0.1812	0.9644	0.0801	0.9514	0.1106
DRRS	0.1150	0.8570	0.3105	0.9690	0.1454	0.9580	0.1979

**Figure 3:**
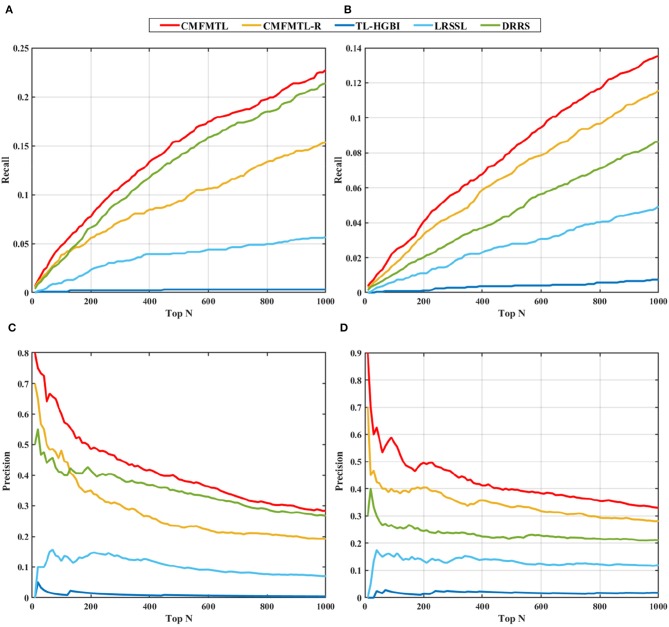
Top-N ranked recall and precision of all methods in two tasks: **(A)** shows the top-N ranked recall in task 1. **(B)** displays the top-N ranked recall in task 2. **(C)** demonstrates the top-N ranked precision in task 1. **(D)** illustrates the top-N ranked precision in task 2.

In practical application, one may be concerned about how many true associations can be recovered by the predictive models from highly ranked predictions. We evaluate the capabilities of all models for top-N predictions. Recall that we randomly select 20% of known therapeutic associations and 20% known non-therapeutic associations, and remove them in 1-fold of 5-CV. We can then investigate the recall scores and precision scores of all models in top predictions ranging from top 10 to top 1,000 (in a step size of 10), and the results are shown in Figure 3. Overall, in both tasks, the proposed CMFMTL method performs best among all methods in terms of both precision and recall at each value of N. Especially, there are more than 50% associations precisely predicted by the CMFMTL within top-100 predictions in both tasks. We ascribe the poor performance of the TL-HGBI to the weak predictive power of the network-based method which heavily relies on the network structure. All the results indicate that CMFMTL absorbs complementary information from two tasks for better performance.

### Case Study

In this section, we use case studies to demonstrate the practical usefulness of CMFMTL in predicting therapeutic and non-therapeutic associations. CMFMTL makes predictions by collective learning, and also shares predictive signals across two tasks. Hence, the prediction scores that the CMFMTL simultaneously generates for two tasks are able to measure the probabilities that drugs associate diseases in a certain association type. We use all drug-disease associations in our dataset to train the CMFMTL model and then rank the prediction scores of all unknown entries which remain unrecorded in the dataset. Then, we focus on the top predicted (drug, disease, association type) triples. We list top 10 ranked predictions in Table 3 and then check up on these associations according to the literature, publications and credible websites. As shown in Table 3, we find evidence to confirm seven associations as well as the corresponding association type. For example, Risperidone, a safe and effective atypical antipsychotic medication, has been frequently used off-label by clinicians to treat Anxiety Disorders (Ravindran et al., 2007). Drug Hypersensitivity is an allergy to a drug and is a form of adverse drug reaction, and the study (Nanau and Neuman, 2010) presented an Ibuprofen-induced clinical manifestation of Hypersensitivity syndrome.

**Table 3:** Top 10 Drug-Disease Associations Predicted by CMFMTL.

**Drug name**	**Disease name**	**Type**	**Evidence**
Chloroquine	Bradycardia	−1	Don Michael and Aiwazzadeh, 1970
Chlorpromazine	Coma	−1	N.A.
Risperidone	Anxiety disorders	1	Ravindran et al., 2007
Clozapine	Headache	−1	https://en.wikipedia.org/wiki/Clozapine
Methotrexate	Neoplasms	1	https://en.wikipedia.org/wiki/Methotrexate
Valproic Acid	Fatigue	−1	N.A.
Amitriptyline	Confusion	−1	https://en.wikipedia.org/wiki/Amitriptyline
Ibuprofen	Drug hypersensitivity	−1	Nanau and Neuman, 2010
Tamoxifen	Diarrhea	−1	N.A.
Vincristine	Neoplasms	1	https://en.wikipedia.org/wiki/Vincristine

*N.A. means that the predicted association cannot be confirmed. Type 1 denotes the therapeutic associations and type −1 refers to non-therapeutic associations*.

## Conclusion

In this work, to simultaneously predict two types of drug-disease association, we present a novel model named collective matrix factorization-based multi-task learning (CMFMTL). Different from existing methods that focus on the existence of drug-disease associations, CMFMTL aims to predict the drug-disease associations and their corresponding association type. Since drug-disease associations are annotated into two categories, predicting each type of association can be served as one individual task. The underlying relatedness across the tasks is a vital piece of prior knowledge that can greatly improve learning abilities. CMFMTL captures the relations between two tasks and successfully utilizes all useful information to achieve high-accuracy and robust performances. The experimental results show that CMFMTL outperforms other state-of-the-art association prediction methods. Case studies demonstrate CMFMTL can find out novel associations and accurately infer the association type.

Nevertheless, CMFMTL still has limitations. CMFMTL predicts the probabilities of therapeutic associations and non-therapeutic associations for all non-interaction drug-disease pairs. However, we notice that some drug-disease associations are included in the top prediction of therapeutic associations as well as the top prediction of non-therapeutic associations. It means that these associations are predicted by CMFMTL to be both therapeutic and non-therapeutic, which is conflicting. The possible reason is that these drugs and diseases are very popular and have a great number of associations. Then, the model learns the data bias. In future work, we will optimize the proposed model to avoid this conflict. Note that similarity integration methods are usually able to achieve high-accuracy performance in similar bioinformatics issues (Zhang et al., 2018d, 2019a,c). We should also consider redesigning our model to integrate several resources of drug feature information.

## Data Availability Statement

Publicly available datasets were analyzed in this study. This data can be found here: The Comparative Toxicogenomics Database (CTD) https://github.com/LoseHair/CMFMTL, DrugBank https://www.drugbank.ca/, Medical Subject Headings (MeSH) http://www.bioinfotech.cn/SCMFDD/.

## Author Contributions

FH and SL designed the project and wrote the manuscript. YQ and QL performed the experiments and analyzed the results. SL and FN supervised and conceived the study. All authors read and approved the manuscript.

### Conflict of Interest

The authors declare that the research was conducted in the absence of any commercial or financial relationships that could be construed as a potential conflict of interest.
